# Effect of Denosumab on Bone Density in Postmenopausal Osteoporosis: A Comparison with and without Calcium Supplementation in Patients on Standard Diets in Korea

**DOI:** 10.3390/jcm12216904

**Published:** 2023-11-02

**Authors:** Chaiho Jeong, Jinyoung Kim, Jeongmin Lee, Yejee Lim, Dong-Jun Lim, Ki-Hyun Baek, Jeonghoon Ha

**Affiliations:** 1 Division of Endocrinology and Metabolism, Department of Internal Medicine, Uijeongbu St. Mary’s Hospital, College of Medicine, The Catholic University of Korea, Seoul 06591, Republic of Korea; cerbere@naver.com; 2 Division of Endocrinology and Metabolism, Department of Internal Medicine, Yeouido St. Mary’s Hospital, College of Medicine, The Catholic University of Korea, Seoul 06591, Republic of Korea; julia@catholic.ac.kr (J.K.); drbkh1@gmail.com (K.-H.B.); 3 Division of Endocrinology and Metabolism, Department of Internal Medicine, Eunpyeong St. Mary’s Hospital, College of Medicine, The Catholic University of Korea, Seoul 06591, Republic of Korea; 082mdk45@gmail.com; 4 Division of General Internal Medicine, Department of Internal Medicine, Seoul National University Bundang Hospital, Seongnam 13620, Republic of Korea; yjlim@snubh.org; 5 Division of Endocrinology and Metabolism, Department of Internal Medicine, Seoul St. Mary’s Hospital, College of Medicine, The Catholic University of Korea, Seoul 06591, Republic of Korea; ldj6026@catholic.ac.kr

**Keywords:** calcium, vitamin D, bone density, postmenopausal osteoporosis, denosumab

## Abstract

The side effects and safety issues tied to calcium supplementation raise questions about its necessity in osteoporosis treatment. We retrospectively evaluated 189 postmenopausal osteoporosis patients treated with denosumab for 12 months. Patients exhibited neither renal dysfunction nor compromised general dietary intake. Patients were divided into three groups as follows: group A, weekly vitamin D 7000 IU; group B, daily vitamin D 1000 IU with elemental calcium 100 mg; and group C, daily vitamin D 1000 IU with elemental calcium 500 mg. All groups showed significant increases in bone density: +6.4 ± 4.7% for the lumbar spine, +2.2 ± 3.5% for the femoral neck, and +2.4 ± 3.8% for the total hip in group A; +7.0 ± 10.9% for the lumbar spine, +2.3 ± 5.2% for the femoral neck, and +2.4 ± 3.8% for the total hip in group B; and + 6.7 ± 8.7% for the lumbar spine, +2.5 ± 8.4% for the femoral neck, and +2.3 ± 4.0% for the total hip in group C. Serum calcium levels increased over time in all three groups with no significant difference. Changes in CTX and P1NP levels did not differ between the groups (all *p* > 0.05). With regular dietary intake, calcium supplementation levels showed no significant effect on bone density, bone marker changes, or hypocalcemia incidence during denosumab treatment.

## 1. Introduction

Osteoporosis is a chronic ailment marked by a reduction in bone density and heightened susceptibility to fractures that influence nearly all parts of the skeletal system [1]. The commonly recommended treatments for osteoporosis include calcium and vitamin D supplementation. However, the use of calcium supplements can lead to constipation and gastrointestinal discomfort and, with long-term use, may increase the risk of kidney stones [2,3,4]. Despite numerous osteoporosis clinical guidelines advocating for calcium and vitamin D supplementation [5,6,7], recent evidence suggests the unfavorable risk–benefit profile of such supplements in osteoporosis treatment [8,9]. Moreover, some studies suggest an increased rate of myocardial infarction, stroke, and sudden death associated with calcium supplementation [10,11,12,13].

The impact of calcium supplements on preventing fractures is also under scrutiny. In a large-scale meta-analysis involving over 52,000 patients, calcium supplements in conjunction with vitamin D showed a significant reduction in hip fracture risk. However, calcium supplements alone did not exhibit the same effect [14]. The RECORD study supports this finding, demonstrating no difference in fracture risk with the use of calcium alone or together with vitamin D [15]. Some studies showed that calcium intake could potentially increase the risk of hip fractures [16,17], which may discourage calcium use. In addition, compliance with calcium supplementation is typically low, possibly affecting adherence to osteoporosis medication [18,19]. Additionally, in clinical practice, it is often challenging to administer calcium supplements for osteoporosis treatment due to side effects like constipation, abdominal discomfort, and heartburn. On the other hand, the administration of vitamin D by itself has few specific side effects; it is, therefore, not uncommon for osteoporosis treatments to be administered without calcium supplementation. Safety and efficacy concerns surrounding calcium supplements necessitate further investigation into the use of vitamin D as a monotherapy for osteoporosis treatment.

Denosumab, a potent antiresorptive agent, is recommended as a first-line treatment for patients with osteoporosis at high to very high risk of fracture in various clinical guidelines. The global use of denosumab is increasing, given its proven effectiveness in significantly increasing bone mineral density (BMD) and reducing fractures. Because of its potent antiresorptive properties, denosumab can lead to hypocalcemia, especially in patients with renal insufficiency. Therefore, it is strongly recommended that patients undergoing denosumab treatment receive a daily supplementation of calcium and vitamin D [20]. However, the risks and side effects tied to calcium supplementation raise questions about its necessity for healthy adults with adequate dietary calcium intake who are receiving denosumab. In clinical practice, when denosumab is administered, and there are adverse effects from calcium supplementation, or if the patient chooses to avoid it, vitamin D might be given on its own, particularly when dietary calcium intake is considered adequate.

In this study, we examined whether BMD and calcium levels vary with denosumab administration based on the amount of calcium supplementation in postmenopausal women with adequate diets.

## 2. Materials and Methods

### 2.1. Study Protocol

We retrospectively evaluated 189 postmenopausal osteoporosis patients under denosumab administration in a tertiary medical center in Korea. The patients did not receive any osteoporosis treatment prior to denosumab. Other than osteoporosis, the patients had no underlying medical conditions such as cancer, autoimmune diseases, or digestive disorders that affect calcium absorption. The questionnaire from the Korean Nutrition Society’s nutrition quotient (NQ) for adults was utilized to determine the patient’s nutritional status [21]. This questionnaire offers a straightforward checklist for outpatient clinics to gauge dietary quality and eating behavior, eliminating the need for a complex dietary survey. Based on a 20-question questionnaire and the nutritional index calculation program from the Korean Nutrition Society, scores were classified as low (0–52.737, below the 25th percentile), medium (52.738–68.482, the 25th to 75th percentile), or high (68.483–100, the 75th percentile or higher), with scores of 58 or more denoting ‘adequate’ nutritional status [21].

Patients were divided into three groups as follows: group A, denosumab and weekly vitamin D 7000 IU (D-MAC^®^, Dalim Biotech, Wonju, Republic of Korea); group B, denosumab and daily vitamin D 1000 IU with elemental calcium 100 mg (DICAMAX D^®^, Dalim Biotech, Wonju, Republic of Korea); and group C, denosumab and daily vitamin D 1000 IU with elemental calcium 500 mg (DICAMAX 1000^®^, Dalim Biotech, Wonju, Republic of Korea). The decision to provide any form of calcium supplementation to a patient was at the discretion of the healthcare provider. In cases of intolerance to calcium supplements, most patients were given vitamin D without calcium supplementation. However, for those with borderline nutritional status and low milk and dairy consumption, formulations with elemental calcium were prescribed. In group A, a weekly dose of 7000 IU vitamin D was prescribed. This was because the only available formulation of vitamin D at our institution was the weekly one. Patients were treated with denosumab 60 mg every six months for 12 months between 2017 and 2019. Although individual dietary calcium intake was not quantified, we checked whether patients were eating a standard diet at the time of the outpatient visit and excluded patients with specific dietary habits such as veganism. As part of a general osteoporosis treatment regimen, it was also recommended that patients consume at least one cup of milk (about 250 mL) daily.

**Figure 1:**
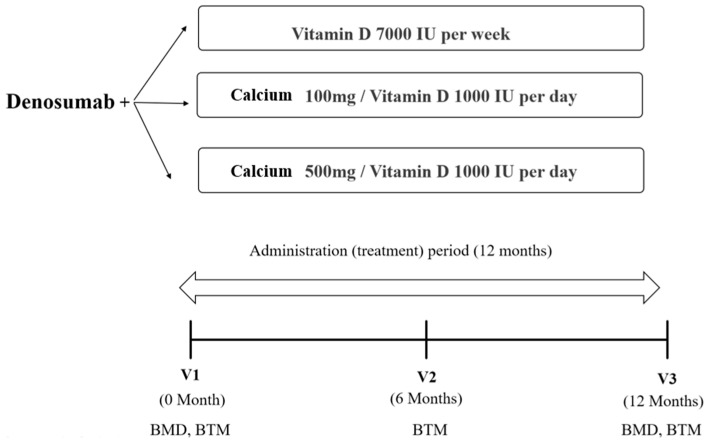
The flow of the study protocol. BMD, bone mineral density; BTM, bone turnover markers.

The BMD (g/cm^2^) values for the lumbar spine, femoral neck, and total hip were measured using dual-energy X-ray absorptiometry (DXA) (Horizon W, Hologic, Inc., Bedford, MA, USA) and analyzed by the same trained technician. The coefficient of variation was 1.0% for the lumbar spine, 1.5% for the femoral neck, and 0.9% for the total hip. Patients underwent DXA every 12 months after the first administration of denosumab. Blood samples were collected after overnight fasting. Bone turnover markers such as serum collagen type 1 cross-linked C-telopeptide (CTX) (Elecsys B-CrossLaps, Roche Diagnostics, Rotkreuz, Switzerland) and procollagen type 1 N-terminal propeptide (P1NP) (Elecsys Total P1NP, Roche Diagnostics) were determined in duplicate using an electrochemiluminescence immunoassay (Cobas e801, Roche Diagnostics). The level of 25-hydroxyvitamin D (25(OH)D) (Access 25(OH) Vitamin D Total DXI reagent, Beckman Coulter, Inc., Brea, CA, USA) was measured using a UniCel DxI 800 Immunoassay Analyzer (Beckman Coulter, Inc.). Serum calcium and phosphorous were assessed before the first dose of denosumab and every six months thereafter. This study was approved by the Institutional Review Board of Seoul St. Mary’s Hospital, The Catholic University of Korea (KC19RISI0846, approved on 7 September 2021). As data were analyzed retrospectively, it was not possible to obtain consent from the participants; hence, this requirement was waived. The study’s research scheme is depicted in Figure 1.

### 2.2. Assessment of the Nutrition Quotient

The NQ, developed by The Korean Nutrition Society, offers a direct evaluation of an individual’s dietary quality and nutritional status [21]. Based on a 20-question survey, scores range from 0 to 100 and are classified as low (below the 25th percentile), medium (25th to 75th percentile), or high (above the 75th percentile) nutritional status. Notably, a score of 58 or above signifies “good” nutritional health. Thanks to its user-friendly format, the NQ is particularly suitable for outpatient contexts, delivering swift assessments without the need for in-depth dietary surveys. After completing the questionnaire, responses are entered into software that calculates the nutrition score. This application is openly available on the Korean Nutrition Society website and can be utilized without requesting permission.

### 2.3. Statistical Analysis

Continuous variables were expressed as the mean ± standard deviation or a percentage unless otherwise stated. To compare the rates of change in clinical markers, descriptive statistics (mean, standard deviation) for each group were used. Multiple comparisons were conducted using analysis of variance (ANOVA), and, where appropriate, Tukey’s Honestly Significant Difference test was applied. The percentage change in BMD was calculated as the absolute change from the baseline to follow-up divided by the baseline value. A two-tailed *p*-value less than 0.05 was statistically significant. All analyses were performed using R software (version 4.0.2, R Foundation for Statistical Computing).

## 3. Results

### 3.1. Baseline Characteristics

The baseline characteristics of patients prior to their treatment with denosumab are summarized in Table 1. The mean age of all patients was 64.9 ± 9.0 years, and there was no difference in age between the three groups. There were no significant differences in the baseline BMI and BMD among the three groups at all measurement sites. The baseline vitamin D level was 23.0 ± 11.8 for group A, 24.6 ± 9.5 for group B, and 23.9 ± 7.3 for group C (*p* = 0.68). The baseline serum calcium level was 8.8 ± 0.5 mg/dL for group A, 8.7 ± 1.1 mg/dL for group B, and 8.9 ± 0.3 mg/dL for group C (*p* = 0.34). The baseline CTX, P1NP, and GFR showed no significant differences between the groups. At the baseline, the nutritional quotient was consistent across the three groups, all of which exhibited good nutritional status.

### 3.2. Changes in BMD during Denosumab Treatment

**Figure 2:**
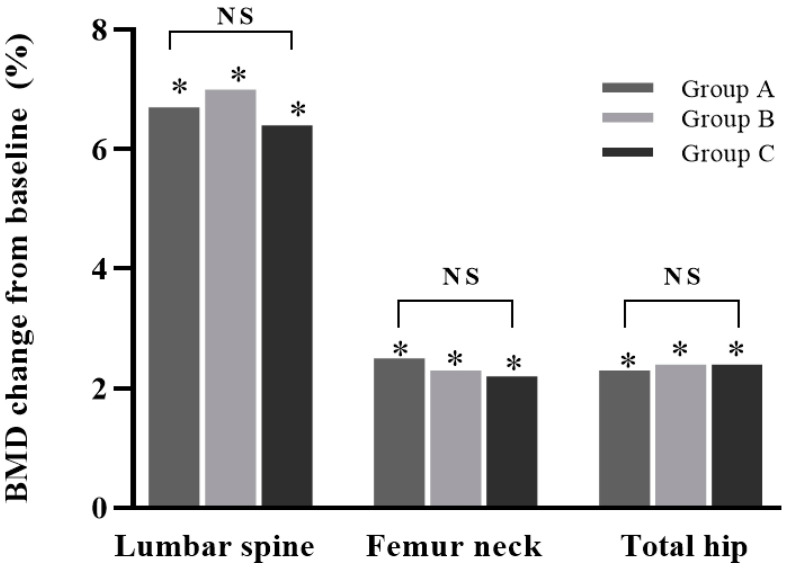
Percentage change in bone mineral density (BMD) from baseline in study groups after 12 months of treatment. Increases in BMD in lumbar spine, femur neck, and total hip were all significant (*, *p* < 0.05). Differences between groups were not significant. NS: not significant (*p* > 0.05). Group A: denosumab and weekly vitamin D 7000 IU alone; Group B: denosumab and daily vitamin D 1000 IU with elemental calcium 100 mg; Group C: denosumab and daily vitamin D 1000 IU with elemental calcium 500 mg.

After 12 months of denosumab treatment, all groups showed significant increases in BMD at all measurement sites compared with the baseline (all *p* < 0.05): +6.4 ± 4.7% for the lumbar spine, +2.2 ± 3.5% for the femoral neck, and +2.4 ± 3.8% for the total hip in group A; +7.0 ± 10.9% for the lumbar spine, +2.3 ± 5.2% for the femoral neck, and +2.4 ± 3.8% for the total hip in group B; and +6.7 ± 8.7% for the lumbar spine, +2.5 ± 8.4% for the femoral neck, and +2.3 ± 4.0% for the total hip in group C. No increase was significantly different between the groups (all *p* > 0.05) (Figure 2).

### 3.3. Changes in Bone Markers during Denosumab Treatment

**Figure 3:**
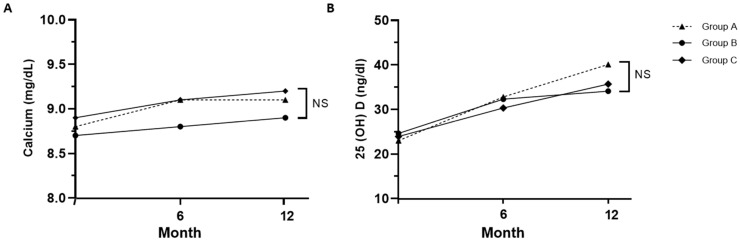
Changes in calcium (**A**) and 25-hydroxyvitamin D (**B**) levels in each group. Serum calcium level was adjusted for albumin; Group A: denosumab and weekly vitamin D 7000 IU; Group B: denosumab and daily vitamin D 1000 IU with elemental calcium 100 mg; Group C: denosumab and daily vitamin D 1000 IU with elemental calcium 500 mg. NS: not significant (*p* > 0.05).

The levels of serum calcium, regardless of the degree of calcium supplementation, and 25-hydroxyvitamin D both increased over time in all three groups during denosumab treatment, and no significant differences in calcium and vitamin D levels were observed between the three groups at 12 months. (Figure 3). In group A, which did not receive calcium supplementation, calcium levels increased from 8.8 ± 0.5 mg/dL at the baseline to 9.1 ± 0.4 mg/dL at 12 months. By the end of 12 months, calcium concentrations were as follows: 9.1 ± 0.4 mg/dL in group A, 8.9 ± 1.2 mg/dL in group B, and 9.2 ± 0.3 mg/dL in group C, which had the highest calcium supplementation. Despite the variations in supplementation, there were no significant differences in calcium concentrations among the three groups (*p* = 0.33). After 12 months, vitamin D concentrations were 40.1 ± 10.3 in group A, 34.1 ± 11.8 in group B, and 35.7 ± 9.9 in group C (*p* = 0.06). Calcium and vitamin D levels significantly increased in all groups at 12 months compared to the baseline; however, no significant differences were noted between the three groups.

**Figure 4:**
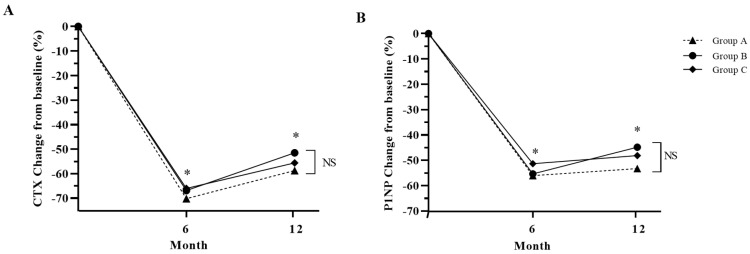
Percentage change in bone turnover markers from baseline in study groups after 12 months of treatment. (**A**): CTX, cross-linked C-terminal telopeptide of type 1 collagen; (**B**): P1NP, total procollagen 1 N-terminal propeptide. Group A: denosumab and weekly vitamin D 7000 IU alone; Group B: denosumab and daily vitamin D 1000 IU with elemental calcium 100 mg; Group C: denosumab and daily vitamin D 1000 IU with elemental calcium 500 mg. *: *p* < 0.05 compared to baseline, NS: Not significant.

The bone markers CTX and P1NP were also suppressed after denosumab treatment for 12 months (Figure 4). Among the three groups in this study, Group A displayed the highest suppression of CTx and P1NP, although there was no statistical difference (Figure 4).

### 3.4. Adverse Events and Safety

In Group A, adverse reactions to supplementation included urticaria in 2 patients (3.2%) and headache in 1 (1.6%). In Group B, 3 patients (4.8%) reported nausea, and 4 (6.3%) experienced constipation. Similarly, in Group C, nausea was reported by 5 patients (7.9%), and constipation was reported by 4 (6.3%). However, none of these adverse events was severe enough to necessitate discontinuation.

## 4. Discussion

In our retrospective study of 189 subjects, we found no differences in BMD and BTM changes among postmenopausal women who maintained an adequate diet, including dietary calcium, regardless of their degree of calcium supplementation during 12 months of denosumab treatment.

The necessity of calcium intake when treating osteoporosis is universally acknowledged. Nonetheless, if side effects arise from calcium supplementation, the practice of preserving dietary calcium while supplementing with vitamin D rather than additional calcium is a significant strategy for enhancing bone density following denosumab administration. Numerous guidelines underscore the significance of enhancing calcium intake through dietary means or supplementation in osteoporosis management [5,6]. Conversely, in alignment with our findings, many studies refute the relevance of calcium intake for preserving bone mass. In a study involving 103 postmenopausal women, the impact of a two-year, 500 mg/day calcium supplement regime on the forearm’s bone mineral content exhibited remarkable similarities across women with diverse baseline calcium intakes [22]. Likewise, evidence from these studies does not uphold the importance of calcium supplementation in maintaining bone mass in the elderly [23]. Furthermore, adding calcium to antiresorptive treatment did not seem to offer any tangible benefits. Bonnick et al. [24] reported that including calcium in alendronate treatment had no impact on BMD. However, there are also studies that highlight the positive impact of calcium on BMD. In a study involving approximately 7000 individuals aged 50 and older, a daily calcium intake below 400 mg was linked to lower BMD, while an intake exceeding 1200 mg/day showed a positive correlation with BMD [25]. Moreover, minor increases in bone density were observed in randomized, controlled trials of calcium supplements, with or without vitamin D [26,27,28].

Our study indicated that a decrease in bone turnover markers was consistent between groups undergoing vitamin D monotherapy and those receiving vitamin D plus calcium supplements. Calcium supplements generally cause a decline in bone turnover markers in postmenopausal women, indicative of improved bone remodeling [29,30,31]. However, the majority of studies evaluate the effect of calcium supplementation in conjunction with vitamin D. Given that vitamin D status is pivotal for calcium absorption, the effectiveness of calcium supplementation alone could differ from that of calcium taken with vitamin D supplements. Therefore, the observed effectiveness of calcium could have been due to the solitary effect of vitamin D. A cautionary note is that administering potent osteoporosis treatments can lead to hypocalcemia, which can be averted with calcium and/or vitamin D supplements. In particular, denosumab is a potent antiresorptive agent, and attention should be paid to the occurrence of hypocalcemia after the administration of this drug. In this study, even among those receiving vitamin D monotherapy, there were no instances of hypocalcemia at 6 or 12 months after treatment. This implies that for patients with a standard diet and no underlying conditions like renal dysfunction, vitamin D supplementation alone could offset denosumab-induced hypocalcemia. It is important to recognize that while calcium supplements have potential drawbacks, they can still offer benefits for certain individuals with osteoporosis. Our study does not refute the beneficial effects of calcium on bone health. However, because of concerns surrounding the side effects of oral calcium supplements and the resulting decrease in medication compliance, we propose that vitamin D monotherapy alone may be sufficient to increase BMD in postmenopausal women with an adequate diet during denosumab treatment. This is further supported by the fact that, in our study, neither nausea nor constipation occurred in the vitamin D-only group. Future research over an extended period is required to substantiate this possibility.

Our study has several limitations. First, we did not quantitatively assess the dietary calcium intake of each participant. In our study, the term ‘adequate diet’ refers to an indirect assessment of nutritional status derived from survey data rather than a quantitative analysis of specific nutrient intake. While the nutrition questionnaire used in this study included an item regarding milk and dairy intake, it did not specify quantities, making it impossible to determine the exact calcium intake for each patient. Although our subjects had no underlying conditions that could hamper calcium absorption and were generally not taking any other medications aside from those prescribed for osteoporosis, we could only estimate their dietary calcium intake. Furthermore, given that patients with similar dietary behaviors are often advised to consume one glass of milk (about 250 mL) daily as part of osteoporosis treatment, significant variations in calcium intake among them are unlikely. A multicenter, cross-sectional study reported that the daily calcium intake in Korean women over 50 years old was 662.8 ± 473.8 mg [32]. The practice of supplementation is highly prevalent among middle-aged Korean women. Hence, considering the additional amount of elemental calcium provided by these supplements along with dietary calcium, it is possible that the recommended daily calcium intake for optimal bone health, which is at least 800 mg, was achieved. Second, due to the restrictions of this retrospective study, we were unable to analyze other biomarkers, such as ALP and PTH, which are valuable when evaluating bone metabolism. Furthermore, hypocalcemia typically occurs within the first month of administering denosumab; however, in common practice settings, it is rare to verify calcium levels within a month of denosumab administration, except in patients with chronic kidney disease. As a result, we lacked data on calcium levels during this critical period. Third, due to the short analysis duration of 12 months, we could not affirm the long-term impact of calcium supplementation on fractures. We could not ascertain if patients independently took additional supplements, such as nutritional aids, during or before denosumab treatment without informing their healthcare provider. Intriguingly, vitamin D levels at the 12-month mark were elevated in group A compared to other groups. This discrepancy could be attributed to the unique dosing of vitamin D in group A, which was 7000 IU weekly, differing from that of the other groups. Another contributing factor could be that patients in group A, who were not prescribed calcium supplements, could have been self-administering other nutritional supplements. Future studies would benefit from considering and closely monitoring such self-supplementation to account for all variables. Lastly, the detection of all fractures within the follow-up interval was problematic. While spinal X-rays, undertaken alongside BMD evaluations, facilitated the identification of any new vertebral fractures, non-vertebral fractures remained undetected in the absence of patient-provided information. Although recognizing fracture incidents is crucial, it was not classified as a significant outcome in this study due to the short research duration.

## 5. Conclusions

Our study observed no difference in BMD changes, alterations in bone markers, or the occurrence of hypocalcemia as a result of the level of calcium supplementation over a 12-month period of denosumab administration in patients on standard diets. Providing that a normal diet is maintained, we anticipated an increase in BMD even in the absence of additional calcium supplementation during denosumab administration. In cases where calcium supplementation is not feasible, it is reasonable to expect the effects of denosumab simply by ensuring an adequate intake of vitamin D. Therefore, these conclusions should be specifically applied to patients who maintain a normal diet with a sufficient dietary calcium intake.

## Figures and Tables

**Table 1:** Baseline clinical and biochemical features of study participants (*n* = 189).

	Group A (*n* = 63)	Group B (*n* = 63)	Group C (*n* = 63)	*p*-Value
Age (years)	64.0 ± 9.8	65.5 ± 9.8	65.3 ± 7.1	0.60
BMI (kg/m^2^)	21.8 ± 2.8	21.3 ± 2.8	21.2 ± 2.9	0.44
Baseline BMD (g/cm^2^)				
Lumbar	0.750 ± 0.067	0.725 ± 0.083	0.744 ± 0.130	0.45
Femur neck	0.543 ± 0.094	0.560 ± 0.085	0.568 ± 0.067	0.40
Total hip	0.671 ± 0.092	0.680 ± 0.086	0.700 ± 0.061	0.29
Baseline T-score				
Lumbar	−2.8 ± 0.7	−2.8 ± 1.1	−2.7 ± 1.0	0.70
Femur neck	−2.6 ± 0.8	−2.6 ± 0.7	−2.7 ± 0.8	0.33
Total hip	−2.0 ± 0.7	−2.1 ± 0.7	−2.2 ± 0.8	0.34
CTX (ng/mL)	0.4 ± 0.4	1.6 ± 7.5	0.4 ± 0.3	0.49
P1NP (ng/mL)	45.5 ± 35.8	43.0 ± 39.4	43.6 ± 19.4	0.96
Calcium (mg/dL)	8.8 ± 0.5	8.7 ± 1.1	8.9 ± 0.3	0.34
Phosphorus (mg/dL)	3.6 ± 0.6	3.8 ± 1.5	3.6 ± 0.4	0.40
25(OH) D (ng/mL)	23.0 ± 11.8	24.6 ± 9.5	23.9 ± 7.3	0.68
Urea nitrogen (mg/dL)	15.5 ± 6.0	16.2 ± 6.8	14.7 ± 4.4	0.51
Creatinine (mg/dL)	0.7 ± 0.2	0.7 ± 0.2	0.6 ± 0.1	0.15
GFR (mL/min/1.73 m^2^)	87.0 ± 19.8	81.5 ± 25.2	91.4 ± 13.2	0.06
Nutrition quotient	62.1 ± 5.4	62.7 ± 6.1	63.1 ± 7.9	0.58

BMI, body mass index; BMD, bone mineral density; CTX, cross-linked C-terminal telopeptide of type 1 collagen; P1NP, total procollagen 1 N-terminal propeptide; 25(OH) D, 25-hydroxyvitamin D; GFR, glomerular filtration rate; the serum calcium level was adjusted for albumin; Group A: denosumab and weekly vitamin D 7000 IU; Group B: denosumab and daily vitamin D 1000 IU with elemental calcium 100 mg; Group C: denosumab and daily vitamin D 1000 IU with elemental calcium 500 mg.

## Data Availability

The data that support the findings of this study are available from the corresponding author, Jeonghoon Ha, upon reasonable request.
